# Eco-friendly voltammetric platform for trace metal determination using a conductive polymer sensor modified with bismuth nanoparticles generated by spark discharge

**DOI:** 10.1007/s00604-023-05929-2

**Published:** 2023-09-02

**Authors:** Alexandra Karapa, Christos Kokkinos, Peter R. Fielden, Sara J. Baldock, Nickolas J. Goddard, Anastasios Economou, Mamas I. Prodromidis

**Affiliations:** 1 Department of Chemistry, National and Kapodistrian University of Athens, 157 71 Athens, Greece; 2 Department of Chemistry, Lancaster University, Lancaster, LA1 4YB UK; 3 Process Instruments (UK) Ltd, March Street, Burnley, BB12 0BT UK; 4 Department of Chemistry, University of Ioannina, 45 110 Ioannina, Greece

**Keywords:** Injection moulding, Electrochemical stripping analysis, Trace metals, Bismuth nanoparticles, Spark discharge

## Abstract

The fabrication of a low-cost eco-friendly sensor platform for the voltammetric determination of trace metals by electrochemical stripping analysis is reported. Plastic conductive electrodes were manufactured via injection moulding from polysterene reinforced with carbon fibres. The platform comprises a carbon counter electrode, a working electrode modified with bismuth nanoparticles generated by spark discharge and a reference electrode coated with AgCl. The sensor fabrication and modification procedures are simple, cost-effective and fast while the materials used are environment-friendly. The utility of the voltammetric platform is demonstrated for stripping analysis of Cd(II) and Pb(II); the limits of detection are 0.7 μg L^−1^ and 0.6 μg L^−1^, respectively (with a deposition time of 240 s) which are comparable to conventional Bi-modified sensors and are sufficient to determine the target metals in water and food samples. The scope of the analytical platform for multi-element assays and for the determination of other trace metals is discussed with representative examples. Therefore, this sustainable and economical platform holds great potential for electrochemical sensing of trace metals.

**Graphical abstract:**

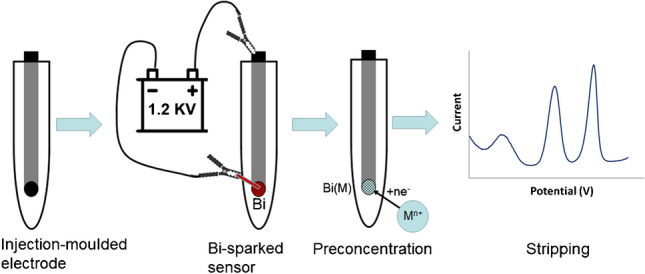

**Supplementary Information:**

The online version contains supplementary material available at 10.1007/s00604-023-05929-2.

## Introduction

Heavy metals are toxic environmental pollutants which can accumulate in biota leading to various health problems [1–3]. In particular, lead and cadmium are strictly regulated in water and food due their potential toxicity [4–7]. Since heavy metals are usually present at low levels in most environmental and food matrices, there is a requirement for sensitive analytical methods for their quantitation. The “golden standard” for the determination of trace metals is atomic spectroscopy in its different variants [8, 9], yet these techniques are mainly laboratory based and labour-intensive and, in addition, require costly equipment and maintenance, trained operators and extensive sample preparation. As a result, despite their advantages as analytical tools, these methods cannot be considered sustainable or economical.

In contrast to atomic spectrometric methods, electrochemical stripping analysis makes use of inexpensive and field-deployable instrumentation and is a convenient technique for on-site assays of trace heavy metals due to its inherent detection sensitivity [10, 11]. The development of innovative sensors for stripping analysis is a major challenge, especially when viewed in conjunction with the (UN) sustainable development goals and the principles of Green Chemistry [12–14]. Diffferent approaches have been pursued for the development of novel sensors, strategies and materials for stripping voltammetry with the view to promote sustainability and eco-friendliness. Major advances in these fields involve the introduction of “green” electrode materials (such as bismuth and antimony) [15–17], the development of sustainable and cost-effective fabrication methods (such as screen printing, 3D printing and mcrofabrication technologies) [18–20] and the implemementation of rapid and field assays [21, 22].

In this work, we describe a new type of sustainable and economical voltammetric platform for trace metal determination. The platform consists of three plastic conductive electrodes manufactured via injection moulding from polysterene reinforced with carbon fibres**.** According to a study comparing the environmental impact of different substrates for screen-printed sensors, plastic exhibited the lowest ecological footprint amongst the material studied (plastic, ceramic, glass, paper and cotton) and is a suitable material for the fabrication of sustainable sensors [23]. On the other hand, injection moulding, in addition to the advantages of high througput, excellent production consistency, high verastility regarding the shape and size of the items created and low cost per unit rate, is also a sustainable fabrication technique since it it involves low labouring costs, produces no waste, does not make use of any chemicals and is applicable to many plastic or hybrid plastic materials most of which are recyclable [24]. The working electrode of the sensor platform is modified with bismuth nanoparticles; bismuth is unique being the only non-toxic heavy metal and this is a main reason that it is classified as a smart “green” material [25–27]. In the present work, the method for the modification of the working electrode with bismuth is based on the formation of bismuth nanoparticles using a spark discharge between a metallic rod and the conductive electrode [28–30]. Spark discharge is also a sustainable technology because it does not make use of chemicals, is highly spatially localized (so that environmental contamination by nanoparticles and waste production are minimal) and is rapid and cost-effective. The use of this approach for the modification of electrodes in electroanalytical chemistry was pioneered by Prodromidis’ and Hrbac’s groups and was sucessfully applied to different organic and inorganic analytes [31–33]. The utility of the electrochemical platform is demonstrated through its application to the determination of Pb(II) and Cd(II) by stripping analysis while its scope to the assay of other target metals is discussed with relevant examples.

## Materials and methods

### Chemicals and reagents

All the chemicals were of analytical grade and purchased from Merck (Darmstadt, Germany). Doubly distilled water was used throughout. Stock solutions containing 10 and 100 mg L^−1^ of different metals (Ca(ΙΙ), Mg(II), K(I), Na(I), Bi(III), Cd(II), Pb(II), Sn(II), Zn(II), In(III), Cu(II) and Tl(I)) were prepared from 1000 mg L^−1^ standard solutions after appropriate dilution with water. A stock 2.0 mol L^−1^ acetate buffer solution (pH 4.5) was prepared from sodium acetate and hydrochloric acid.

### Instrumentation-software

For electrochemical measurements, a portable EmStat potentiostat was used (Palmsens, Houten, the Netherlands). Control, data acquisition and data evaluation were performed with the PSTrace 5.5 software. A stirrer (Speedsafe, Hanna) was used to provide solution agitation by means of a star-shaped stirring bar rotating at 1000 rpm.

The sparking process was carried out using a high-voltage power supply fabricated in-house [31].

An injection moulding machine (Babyplast 6/6, Cronoplast SA, Barcelona, Spain) was used to fabricate the plastic electrodes.

Scanning electron microscopy (SEM) images were obtained with a JEOL JSM6510LV scanning electron microscope equipped with an INCA PentaFETx3 (Oxford Instruments) energy dispersive X-ray (EDX) spectroscopy detector (Microscopy Unit of the University of Ioannina).

 The mean bismuth particle size was calculated form the image of the EDX mapping for bismuth using the open-access Image J v.1.54d software (https://imagej.net/ij/).

### Preparation and modification of the voltammetric platform

**Fig. 1:**
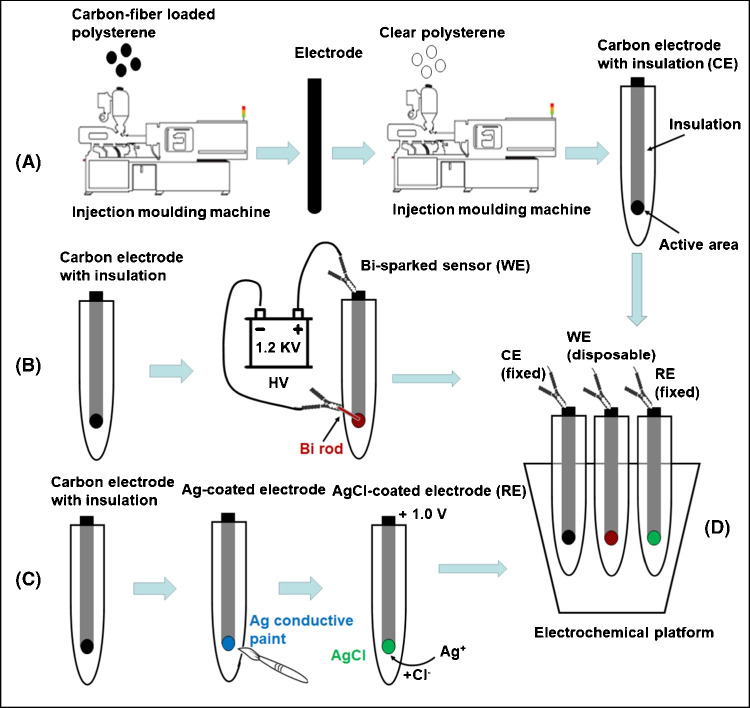
Schematic diagram of the fabrication process of (**A**) the CE, (**B**) the WE and (**C**) the RE. (**D**) Schematic diagram of the injection-moulded electrochemical platform. WE is the working electrode, RE is the reference electrode, CE is the counter electrode and HV is a high-voltage power supply

The conductive electrodes were first injection-moulded from 40% carbon fibre-loaded polystyrene (RTP 487, RTP Company, Bury, UK) and then encased into an insulating matrix of clear non-conductive polystyrene (Northern Industrial Plastics Ltd., Chadderton, UK) by overmoulding leaving a circular area exposed (2.5-mm diameter) which serves as the active surface of the electrode (Fig. 1A). The electrodes were manufactured in arrays of 5 (Fig. S1, Supplementary Material). The average fabrication time of an array of 5 sensors is 30 s (5–7 s for injection moulding of the conductive electrodes and 20–30 s for overmoulding in clear plastic).

The process of modification of an injection-moulded electrode with bismuth nanoparticles for the formation of the working electrode is illustrated in Fig. 1(B). A bismuth rod (fabricated as described in the Supplementary Material) was connected to the cathode ( −) and the electrode to the anode ( +) of the high-voltage power supply; the sparking process was initiated by bringing the bismuth rod in contact with the electrode and sweeping uniformly the whole active area of the electrode. The reference electrode was formed by coating the working surface of the injection-moulded electrode with a Ag-based conductive paint (Granville Electro Connector, Granville Oils & Chemicals Ltd, Goldthorpe, UK) and further coating with AgCl by connecting the electrode as the working electrode in a three-electrode configuration and applying + 1.0 V in a cell filled with 0.1 mol L^−1^ KCl for 10 s (Fig. 1(C)). The electrochemcial platform is composed of the bare carbon electrode as the counter electrode (CE), the Bi-sparked electrode as the working electrode (WE) and the Ag/AgCl-coated electrode as the reference electrode (RE) (Fig. 1(D)); the CE and RE of the platform are re-usable while the WE is disposable.

### Samples

The honey sample was purchased from a local supermarket. 1.0 g of the honey sample was accurately weighed and was digested in a hotplate for 20 min in 5 mL HCl (30%) and 1 mL H_2_O_2_(30%) almost to dryness. The solution was brought to a final volume of 10 mL with addition of 0.1 mol L^−1^ acetate buffer (pH 4.5), 10 μL of a 1.0 × 10^−^^2^ mol L^−1^ K_4_[Fe(CN)_6_] solution was added (to achieve a final 1.0 × 10^−5^ mol L^−1^ concentration of K_4_[Fe(CN)_6_]) and the sample was analyzed as described in the “Experimental Procedure” section. A spiked honey sample was prepared by spiking 1.0 g of accurately weighed honey sample with 10 μL of the 10 mg L^−1^ stock Pb(II) solution and 5 μL of the 10 mg L^−1^ stock Cd(II) solution and was treated and analyzed as above. The final concentrations in the treated sample were 10 μg L^−1^ Pb(II) and 5 μg L^−1^ Cd(II). Quantitation was performed with two standard additions, each consisting of 5 μL of the 10 mg L^−1^ Cd(II) stock solution and 10 μL of the 10 mg L^−1^ Pb(II) stock solution.

The drinking water sample was collected from a local house tap. 9.5 mL of the sample was mixed with 0.5 mL of 2 mol L^−1^ acetate buffer (pH 4.5), 10 μL of a 1.0 × 10^−^^2^ mol L^−1^ K_4_[Fe(CN)_6_] solution was added (to achieve a final 1.0 × 10^−5^ mol L^−1^ concentration of K_4_[Fe(CN)_6_]) and the solution was directly analyzed as described in the “Experimental Procedure” section. A spiked drinking water sample was prepared by collecting 9.5 mL of water from a local house tap, mixing with 0.5 mL of 2 mol L^−1^ acetate buffer (pH 4.5) and spiking with 10 μL of the 10 mg L^−1^ stock Pb(II) solution and 10 μL of the 10 mg L^−1^ stock Cd(II) solution before further treatment and analysis as described above. The final concentrations in the treated sample were 10 μg L^−1^ Pb(II) and 10 μg L^−1^ Cd(II). Quantitation was performed with two standard additions, each consisting of 10 μL of the 10 mg L^−1^ Cd(II) stock solution and 10 μL of the 10 mg L^−1^ Pb(II) stock solution.

### Experimental procedure

Cyclic voltammograms (CVs) with the voltammetric platform were obtained by placing the three electrodes in a cell containing 0.1 mol L^−1^ acetate buffer (pH 4.5), connecting to the potentiostat and scanning the potential from − 1.4 to + 0.2 V and back again at a scan rate of 50 mV s^−1^.

The experimental procedure for the determination of Pb(II) and Cd(II) by square wave anodic stripping voltammetry (SWASV) with the voltametric platform is schematically illustrated in Fig. S2. 10 mL of the sample was transferred to the cell, and the three electrodes were immersed in the sample and connected to the potentiostat. Preconcentration took place by applying a potential of − 1.2 V for 240 s to the WE under stirring. The stirring was stopped and the solution was left to equilibrate for 5 s. Then, a square wave anodic scan was applied to the WE from − 1.2 to − 0.3 V (frequency, 50 Hz; step, 4 mV; pulse height, 20 mV) which caused the accumulated target metals to oxidize. The anodic voltammogram was recorded and the stripping peak heights (in terms of oxidation current) were measured for quantification purposes. At the end of each preconcentration/stripping period, the WE was cleaned for 20 s by applying a potential of − 0.3 V under stirring. All the measurements were carried out in the presence of dissolved oxygen.

For the multi-element determination of Cd(II), Pb(II) and Zn(II), preconcentration took place by applying a potential of − 1.4 V for 240 s to the WE. For the determination of Tl(I) in the presence of multivalent cations, the sample was spiked with 2 × 10^−5^ mol L^−1^ of EDTA.

The experimental procedure for the determination of Pb(II) and Cd(II) by square wave anodic stripping voltammetry (SWASV) at an in situ Bi-electroplated electrode  was similar to the procedure described above. In this case, a bare plastic electrode was used as the WE, the sample was spiked with 2 mg L^−1^ Bi(III) and the square wave anodic scan was applied to the WE from − 1.2 to + 0.2 V.

## Results and discussion

### Characterization of the Bi-sparked working electrode

**Fig. 2:**
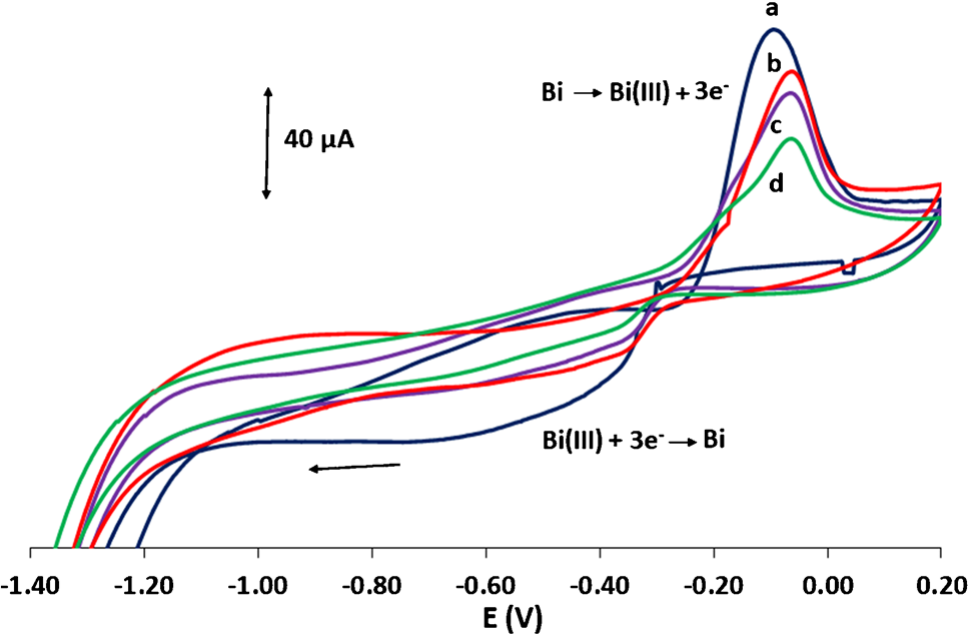
Four successive CV scans of a Bi-sparked WE in acetate buffer (pH 4.5) in the range − 1.4 tο + 0.2 V. Scan rate, 50 mV s.^−1^

The deposition of the bismuth on the plastic WE was studied by electrochemical and optical techniques. Repetitive CVs using a freshly prepared Bi-sparked WE in acetate buffer (pH 4.5) in the range − 1.4 tο + 0.2 V are illustrated in Fig. 2. The first anodic forward scan (Fig. 2a) produced a sharp peak at − 0.07 V which is attributed to the oxidation of the bismuth nanoparticles to Bi(III) while the first cathodic reverse scan  gave rise to a wide reduction peak which is attributed to the partial reduction of the Bi(III) generated at the forward scan back into metallic bismuth. Subsequent repetitive CV scans (Fig. 2b to d) gave rise to continuously weaker oxidation and reduction signals suggesting that the Bi was eventually depleted from the WE surface during the successive oxidation-reduction processes. This is presumably due to the fact that only part of the Bi(III) formed during the oxidation processes redeposited on the WE as bismuth. A practical implication of this effect is that the potential of the WE must be maintained at potentials rather more negative than the stripping potential of bismuth in order to preserve the bismuth coating on the surface of the WE and to achieve reproducible results. Thus, an anodic limit of − 0.3 V was set for experiments involving the Bi-sparked WE.

**Fig. 3:**
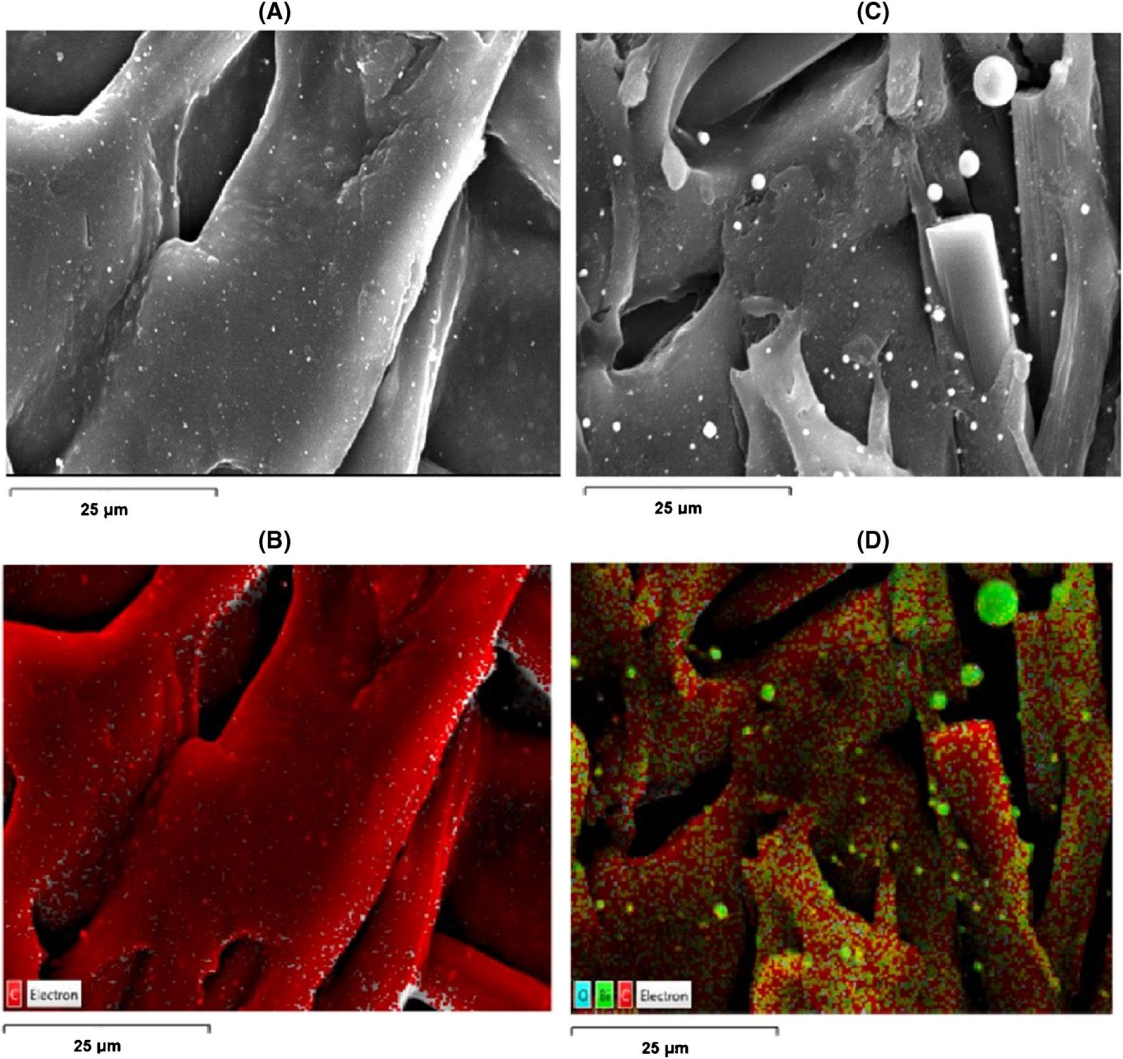
**(A)** SEM and **(B)** EDX mapping for carbon at a bare conductive electrode. **(C**) SEM and (**D)** EDX mapping for carbon, oxygen and bismuth at a conductive electrode coated with bismuth nanoparticles using spark discharge

The distribution of the bismuth nanoparticles on the surface of the Bi-sparked WE was studied by SEM and mapped by EDX. At the carbon fibre–loaded electrode, the carbon fibres embedded in the polymer matrix are clearly visible in the SEM image (Fig. 3(A)) and are visually identified by the EDX mapping for carbon (red colour in Fig. 3(B)). After sparking the WE with bismuth, the SEM image reveals the formation of nanoparticles (Fig. 3(C)) which are clearly visible on the EDX mapping (Fig. 3(D)). The data in Fig. 3 suggest a uniform distribution of bismuth nanoparticles (identified by the green colour on the EDX mapping) at the electrode surface with only a minimal degree of aggregation; the average size of the bismuth nanoparticles was calculated as 220 ± 50 nm.

While it has been suggested previously that bismuth could deposit as bismuth oxide due to the high temperature prevailing during the sparking process in the presence of oxygen [31], the EDX spectra before and after sparking (Fig. S3 (Supplementary Material)) indicate that the sparking process does not cause quantifiable change of the oxygen content at the electrode surface. In addition, the poor correlation of the EDX surface mapping data for oxygen and bismuth at the sparked electrode does not support deposition of bismuth as its oxide (Fig. S4 (Supplementary Material)).

### Comparison between sparking and in situ bismuth electroplating

**Fig. 4:**
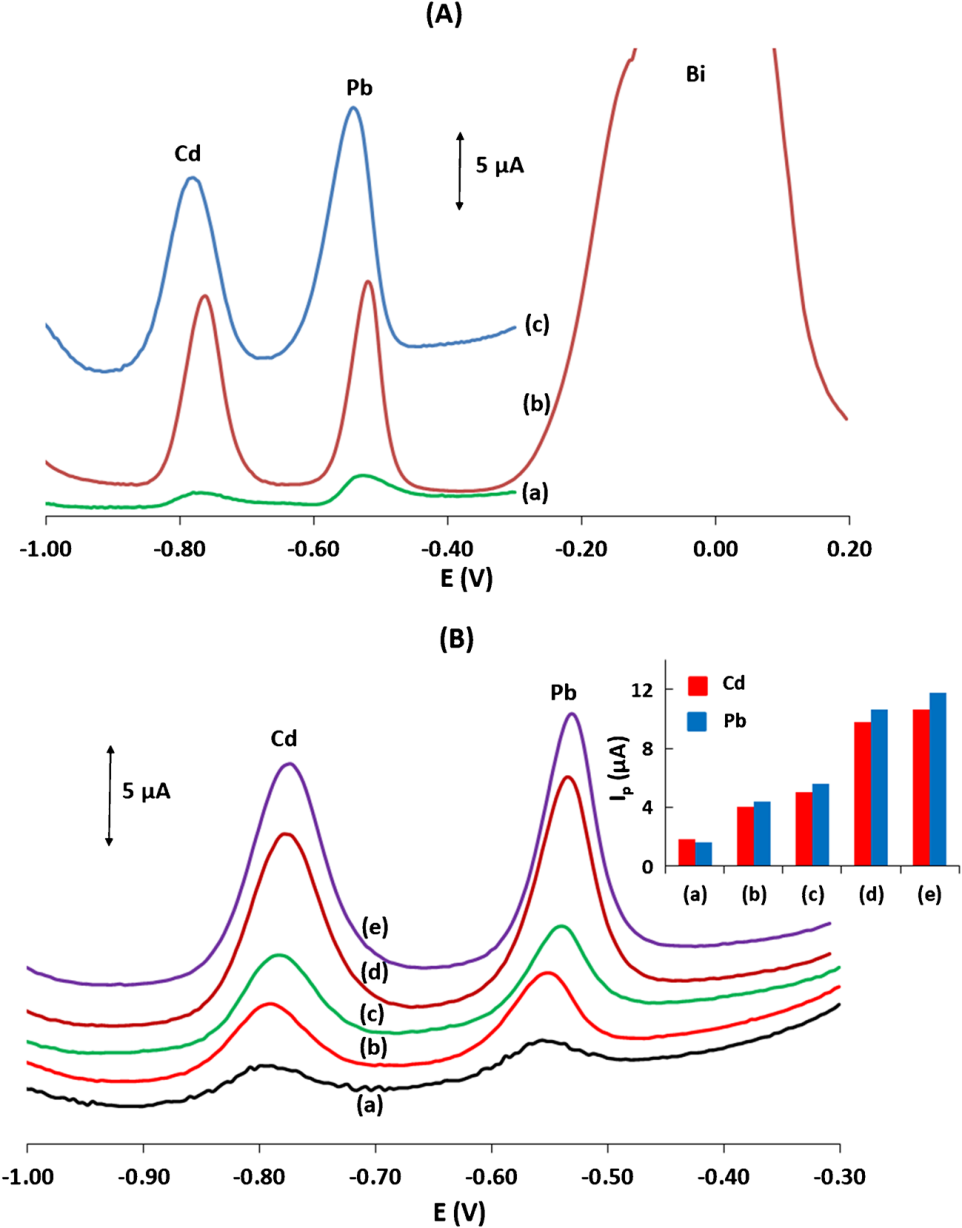
**(A**) Comparison between: (a) a bare conductive WE, (b) an in situ Bi-electroplated WE, (c) a Bi-sparked WE, for the determination of 40 μg L^−1^ Pb(II) and Cd(II) by SWASV. **(B)** Effect of the polarity and the sparking voltage applied to the conductive WE on the SWASV response of 40 μg L^−1^ Pb(II) and Cd(II): (a) bismuth rod as anode ( +) and sparking voltage 1000 V, and (b–e) bismuth rod as cathode ( −) and sparking voltage 400, 600, 1000 and 1200 V, respectively. Peak heights are shown in the insert. Deposition potential, − 1.2 V; deposition time, 240 s; supplementary electrolyte, 0.1 mol L^−1^ acetate buffer (pH 4.5)

The most common method for modification of electrodes with a bismuth film in stripping analysis is in situ electroplating, i.e. deposition of a bismuth film by reduction of Bi(III) added in the sample solution [15–17]. Therefore, a comparison was made between the stripping response for Pb(II) and Cd(II) obtained at a plastic conductive Bi-sparked WE and an in situ Bi-electroplated plastic conductive WE. As illustrated in Fig. 4(A)(b and c), the two Bi-coated WEs produced comparable sensitivity with the in situ Bi-electroplated WE yielding narrower peaks; this is attributed to the “thinner” films formed by the electroplating method which facilitate faster diffusion of the stripped species from the working electrode. However, sparking is a “greener” method because it does not make use of chemicals and does not produce waste. In addition, since sparking can produce ready-to-use sensors, it is more convenient for on-site analysis where a simple analytical protocol is desirable. As expected, the modification of the electrode with bismuth (irrespective of the method) greatly enhanced the sensitivity compared to a bare plastic conductive working electrode (Fig. 4(A)(a)).

### Selection of the sparking voltage and its polarity

The sparking voltage is expected to have a profound effect on the modification of the WE with bismuth nanoparticles [31–33]. The sparking voltage was varied in the range 400–1200 V by connecting the bismuth rod to the cathode ( −) of the high-voltage power supply (Fig. 4(B)(b to e)). The stripping responses of Pb(II) and Cd(II) at WEs sparked at different voltages increased as the voltage was raised from 400 to 1000 V and increased only marginally at higher voltages (Fig. 4(B) (insert)). Therefore, a sparking voltage of 1200 V was used. On the other hand, reversing the polarity of the sparking process by connecting the bismuth rod to the anode ( +) of the high-voltage power supply produced a weak and noisy stripping signal (Fig. 4(B)(a)).

### Selection of the supporting electrolyte and the stripping waveform

Four supporting electrolytes were investigated (0.1 mol L^−1^ HCl, 0.1 mol L^−1^ HNO_3_, 0.1 mol L^−1^ H_2_SO_4_ and 0.1 mol L^−1^ acetate buffer (pH 4.5)) which were compared for the ASV determination of Pb(II) and Cd(II) (Fig. S5(A) (Supplementary Material)). Only in the case of the acetate buffer were well-defined peaks for the target metals obtained and this observation agrees with previous studies in which acetate buffers with pH 4–5 are recommended and prefered over more acidic media [15–17].

Another crucial factor that affects the reponse in ASV is the waveform used for the stripping step, in terms of sensitivity, background correction and rapidity. Three common potential-time waveforms are investigated for performing the stripping step: linear sweep (LS), differential pulse (DP) and square wave (SW). The LS mode is fast and offers reasonable sensitivity but does not discriminate well against against capacitive current and the background of the voltammogram is slopping (Fig. S5(B-a) (Supplementary Material)). The DP mode provided a flat baseline but its sensitivity was low and is limited to slow scan rates so that the analysis time is longer (Fig. S5(B-b) (Supplementary Material)). The square wave mode provided excellent background characteristics and sensitivity combined with rapidity (Fig. S5(B-c) (Supplementary Material)); thus, it was selected for the rest of the experiments.

### Selection of the accumulation parameters

The main deposition parameters are the deposition potential and the deposition time and these conditions can be judiciusly selected depending on the sensitivity required. The deposition time was varied in the interval 0 to 600 s (Fig. S6(A) (Supplementary Material)). The stripping peak heights for Pb and Cd increased with increasing deposition time following a second-order pattern in which the rate of increase was more pronounced at lower deposition times and dropped at higher deposition times. For the experiments in this work, a deposition time of 240 s was selected but higher deposition times can be selected for higher sensitivity at the expense of a longer analysis time. The deposition potential was studied in the interval − 0.6 to − 1.6 V (Fig. S6(B) (Supplementary Material)). The stripping peak heights for Pb and Cd increased as the deposition potential became more negative since the deposition efficiency is favoured at higher carthodic overpotentials; at deposition potentials more negative than − 1.0 V, the stripping peak heights were not much affected since conditions of mass-transfer cotrol were established in this region. A deposition potential of − 1.2 V was selected for the rest of this work.

### Metrological data

**Fig. 5:**
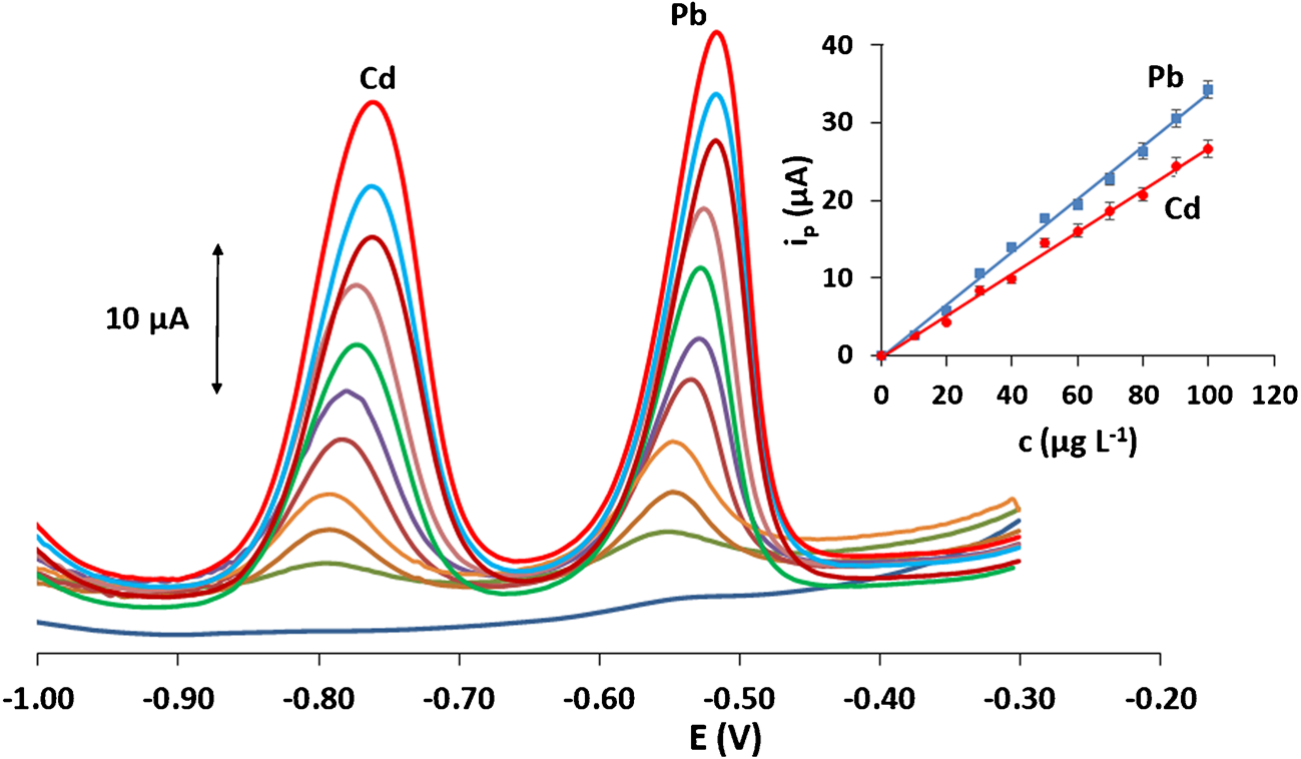
SW stripping voltammograms for the determination of Pb(II) and Cd(II) in the concentration range 10–100 μg L^−1^ (calibration plots as insert) at the voltammetric platform in 0.1 mol L^−1^ acetate buffer (pH 4.5) (the bottom voltammogram is the blank). The voltammograms at the bottom were obtained when no target metals were present in solution. Deposition at − 1.2 V for 240 s; other conditions as in the Experimental Part

SW stripping voltammograms of Cd(II) and Pb(II) were obtained in 0.1 mol L^−1^ acetate buffer (pH 4.5). The calibration curves for the target metals were linear (Fig. 5, insert) with an analytical sensitivity of 0.26 μΑ μg^−1^ mL for Cd(II) and 0.33 μΑ μg^−1^ mL for Pb(II). The limits of detection (LODs) were 0.7 μg L^−1^ for Cd(II) and 0.6 for Pb(II) (calculated using the equation LOD = 3 × *s*_*xy*_/*S* (where *s*_*xy*_ is the standard deviation of the residual of the calibration plot and *S* is the slope of the calibration plot)) [34]. The analytical and fabrication features of the bismuth-sparked injection-moulded sensors were compared with those of existing carbon/polymer electrochemical sensors and of those of bismuth-modified disposable electrodes used for Pb(II) and Cd(II) determination (Table S1, Supplementary Material). The LODs are amongst the lowest reported so far for both carbon/polymer and bismuth-modified disposable electrodes at comparable deposition times. In addition, injection moulding provides a much higher fabrication throughput than 3D printing (several seconds as opposed to several minutes, respectively) and compares favourably with the dominant technology of screen-printing in terms of scope for mass fabrication without requiring chemicals or producing waste. Additionally, sparking provides a simpler and faster electrode modification protocol with bismuth than the commonest methods involving electroplating with metallic bismuth or bulk-modification with bismuth precursors.

An important parameter is the precision achieved using the voltammetric platform. The within-electrode repeatability was calculated by carrying out 8 complete stripping cycles at the same Bi-sparked WE electrode lasting in total 36 min. At 40 μg L^−1^ of Pb(II) and Cd(II), the % relative standard deviations of the stripping peak heights were 4.5% for Pb(II) and 5.1% for Cd(II) while at 10 μg L^−1^ of Pb(II) and Cd(II), the % relative standard deviations of the stripping peak heights were 5.8% for Pb(II) and 7.1% for Cd(II). The within-electrode repeatability was satisfactory and this is attributed to the cleaning step of the WE at − 0.3 V that eliminates carry-over effects between measurements and the strict control of the WE potential at potential more cathodic than − 0.3 V that prevents the oxidation, and thus preserves the integrity, of the bismuth deposit. For routine measurements, a single WE was used for a series of 3–10 measurements (e.g. to perform a complete calibration or analysis using standard additions). The between-electrode reproducibility was calculated by carrying out complete stripping cycles at 6 different Bi-sparked WEs. At 40 μg L^−1^ of Pb(II) and Cd(II), the % relative standard deviations of the stripping peak heights were 9.5% for Pb(II) and 11.3% for Cd(II). As expected, the between-electrode reproducibility was lower than the within-electrode repeatability and this is attributed to the differences in the active electrode surface between electrodes and the different morphologies of the bismuth deposit at different electrodes.

### Interferences and scope for multielement determinations

Different cations (Sn(II), Zn(II), Sb(III), Tl(I), In(III), Zn(II) and Cu(II)) that are expected to accumulate on the Bi-sparked WE and interfere with the determination of Cd(II) and Pb(II) were assessed (Fig. S7, Supplementary Material). Sn(II) and Sb(III) did not give rise to stripping peaks in the potential range used in this work and did not interfere. In(III) produced a strong stripping peak at − 0.71 V that severely overlapped with the Cd peak and Tl(I) yielded a weak peak at − 0.64 V that lied between the Cd and Pb peaks. However, in the vast majority of environmental and food samples, Tl and In are found at ultratrace concentrations, much lower than those of Cd(II) and Pb(II) [35, 36]. Zn(II) produced a stripping peak at − 1.14 V only when a preconcentration potential more negative than − 1.3 V was applied to the WE. Cu(II) did not give rise to a stripping peak within the potential range used but induced severe suppression of the Cd and Pb peaks; this was the most serious interference because Cu(II) is ubiquitous and its interference cannot be easily addressed by instrumental means (e.g. control of the deposition potential) since Cu co-deposits with Cd and Pb. In this work, a chemical approach was used to alleviate the Cu(II) interference, namely the addition of ferrocyanide as recommended earlier [37]. Other cations, such as Ca(ΙΙ), Mg(II), K(I), Na(I), Cl^−^, NO_3_^−^ and SO_4_^2−^, added at a 100-fold excess over Pb(II) and Cd(II) did not interfere. Another common interference is the presence of organic surfactants that can adsorb on the working electrode causing fouling and inactivation of its surface [38]; Triton X-100 was used as a model surfactant to simulate this effect. 0.5, 1, 2, 5 and 10 mg L^−1^ of Triton X-100 caused reduction of the Cd and Pb peaks by 5, 12, 37, 56 and 89% and 8, 17, 45, 71 and 95%, respectively, indicating that a pre-treatment step is necessary in surfactant-rich samples.

**Fig. 6:**
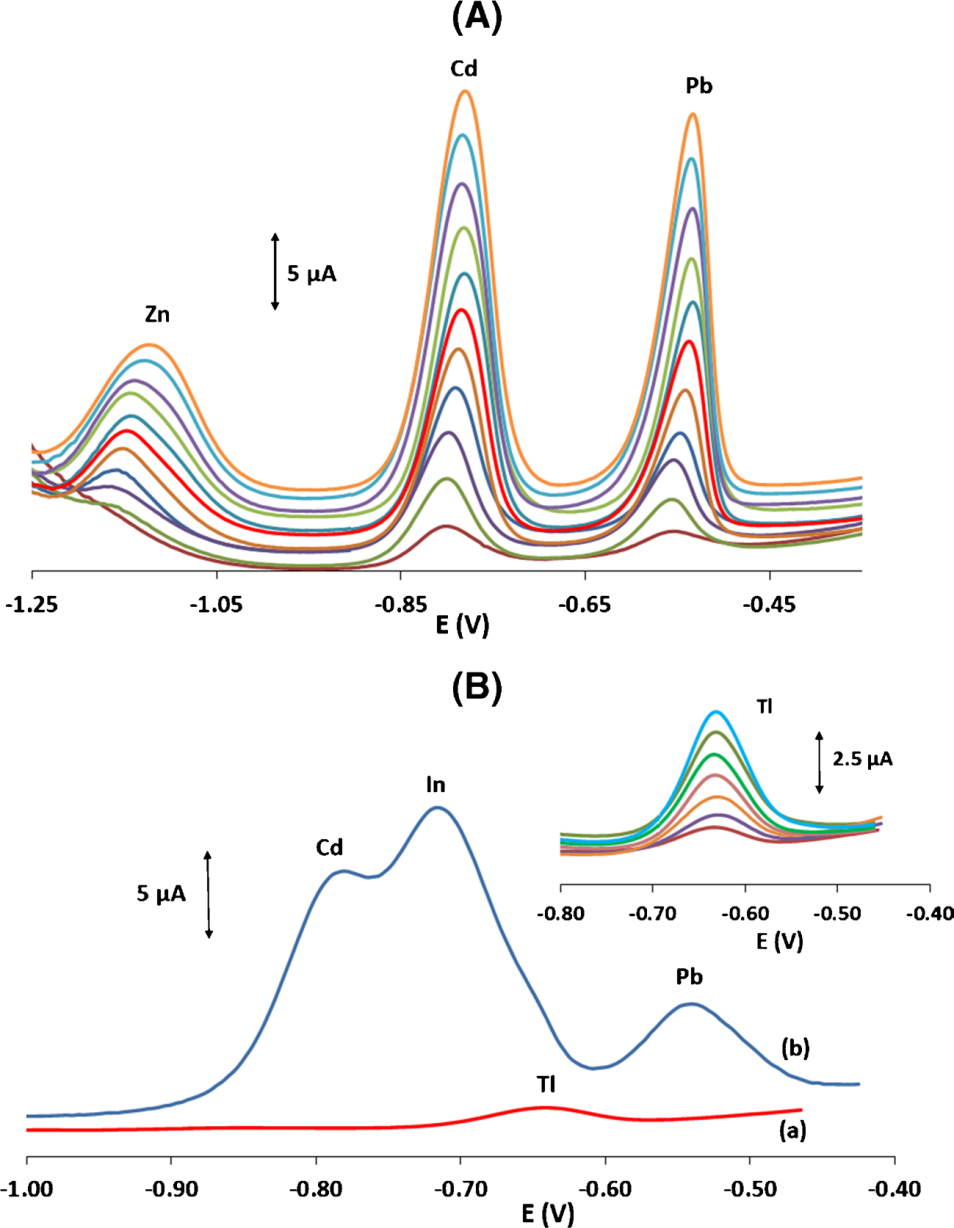
**(A)** SW stripping voltammograms for the simultaneous determination of Zn(II), Cd(II) and Pb(II) in in the concentration range 10–110 μg L^−1^. Deposition potential, − 1.4 V. **(B**) SW stripping voltammograms for (a) 10 μg L^−1^ T(l) and (b) 10 μg L^−1^ T(l) in the presence of 40 μg L^−1^ each of Cd(Il), In(III), Pb(II) and Cu(II). Insert: SW stripping voltammograms for 10–70 μg L^−1^ T(l) in the presence of 40 μg L^−1^ each of Cd(Il), In(II), Pb(II) and Cd(II) and 2 × 10^−5^ mol L^−1^ EDTA

The scope of the Bi-sparked sensors for the determination of additional metal cations was also investigated with two representative examples. The first example is the possibility of performing multi-element determinations by judicious adjustment of the instrumental conditions. Figure 6(A) illustrates stripping voltammograms for the simultaneous determination of Zn(II), Cd(II) and Pb(II) using a deposition potential of − 1.4 V, demonstrating the satisfactory linearity achieved for the three target metals. The second example refers to the determination of Tl(I) in the presence of Cd(II), In(III) Pb(II) and Cu(II), while Tl(I) itself gives rise to a well-defined stripping peak (Fig. 6(B)(a)); addition of the aforementioned cations results in stripping peaks that interfere strongly with, and almost totally shadow, the weak Tl(I) stripping peak (Fig. 6(B)(b)). Addition of EDTA in the solution leads to the formation of complexes with the multi-valent cations but not with the monovalent Tl(I), so that determination of Tl(I) is feasible as illustrated in Fig. 6(B) (insert) [39].

### Applications

The voltametric platform was applied to the determination of Pb(II) and Cd(II) in a honey sample and a drinking water sample. The sample treatment is described in the “Samples” section and the analysis protocol is described in the “Experimental Procedure” section. Since in both samples the Pb(II) and Cd(II) concentrations were lower than the limit of detection, the accuracy was determined by spiking the samples with target metals and applying the method of multiple standard additions as described in the “Samples” section. The recovery, *R* %, was calculated using the formula:

$$R\mathrm{ \%}=\left({C}_{\mathrm{exp}}/{C}_{\mathrm{sp}}\right)\times 100$$

where *C*_exp_ is the target concentration experimentally determined by the method of standard aditions and *C*_sp_ is the spiking concentration.

**Fig. 7:**
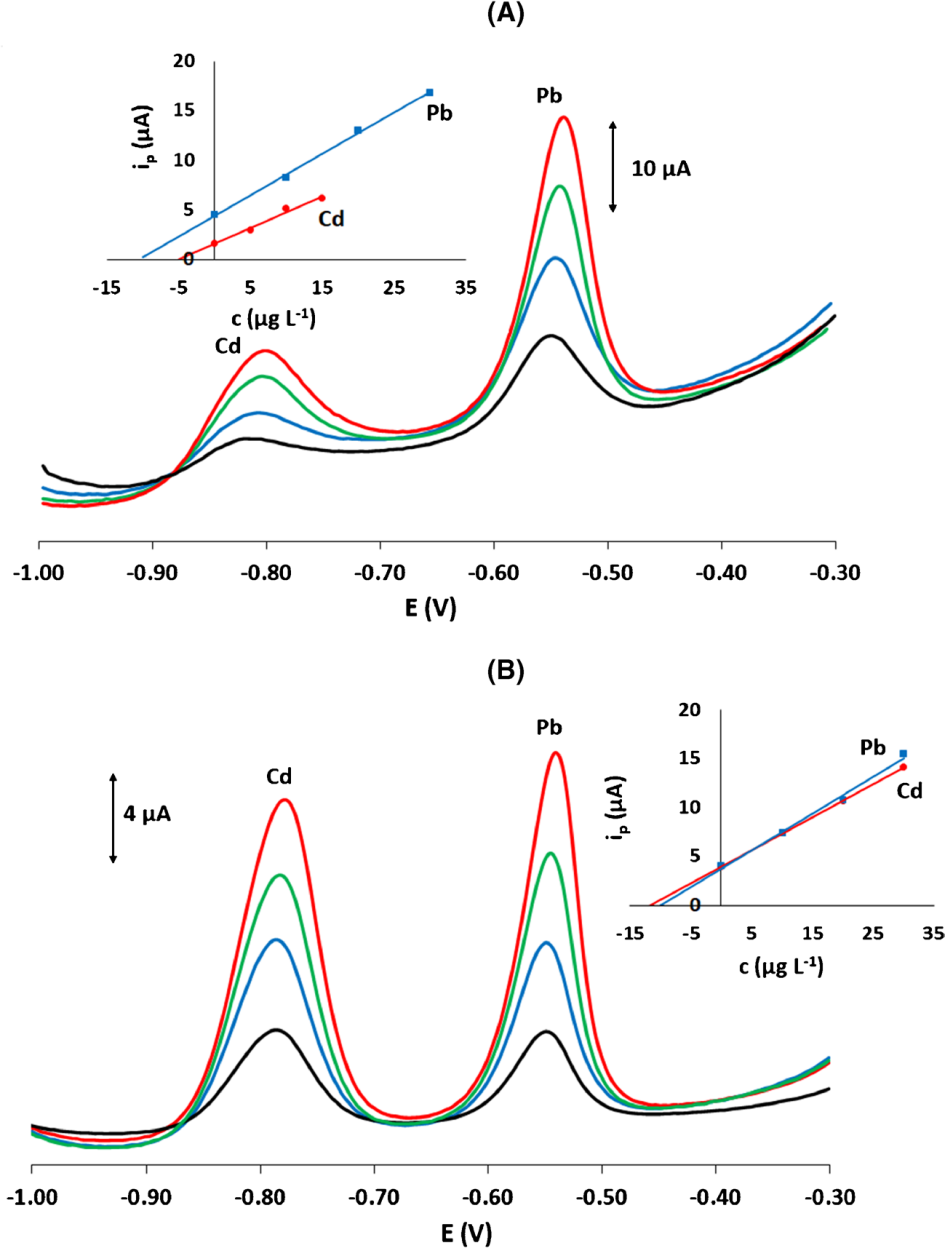
SW stripping voltammograms for the determination of Pb(II) and Cd(II) in (**A**) a honey sample and (**B**) a tap water sample using the method of standard additions. Standard addition plots are shown as inserts

For the honey sample, spiking with Pb was performed at 0.1 mg/kg (which is the maximum level regulated by the EU [7]) and with Cd to 0.05 mg/kg (this was set provisionally lower than the Pb level since no limit for Cd is set by the EU [6]). The method of standard additions was used to determine the Pb(II) and Cd(II) concentrations in the spiked-treated sample. The voltammograms for the spiked sample and after standard additions of Pb(II) and Cd(II) are illustrated in Fig. 7(A) and the standard addition plot is shown as an insert. The recoveries were 99 ± 10% for Cd and 105% ± 8 for Pb (*n* = 3).

For the drinking water, spiking was performed at 10 mg L^−1^ for Pb(II) and Cd(II) to simulate a non-compliant sample containing low concentrations of the target metals (the permissible limit is 5 mg L^−1^ for both Pb(II) and Cd(II) [40]). The method of standard additions was used to determine the Pb(II) and Cd(II) concentrations in the treated sample. The voltammograms for the spiked sample and after standard additions of Pb(II) and Cd(II) are illustrated in Fig. 7(B) and the standard addition plot is shown as an insert. The recoveries were 99 ± 10% for Cd and 105% ± 8 for Pb (*n* = 3).

## Conclusions

In this work, we describe an eco-friendly voltammetric platform for trace metal analysis. The materials and fabrication methodology are sustainable involving low-cost, low ecological footprint and non-toxic “green” materials. Analysis was performed using a plastic conductive WE coated with bismuth nanoparticles by spark discharge. Proof-of-concept application of the platform was demonstrated for the determination of Pb(II) and Cd(II) while the scope for assays of other trace metals is discussed. The voltammetric platform is suitable for field analysis and the metrological data indicate the methodology is fit-for-purpose for the analysis of water and food samples.

### Supplementary Information

Below is the link to the electronic supplementary material.

Supplementary file1 (1.06 MB)

## References

[1] Masindi V, Muedi K (2018) Environmental contamination by heavy metals. In: El-Din H; Saleh M, Aglan RF (ed) Heavy metals, Intech Open. 10.5772/intechopen.76082

[2] Azeh Engwa G, Udoka Ferdinand P, Nweke Nwalo F, Unachukwu MN (2019) Mechanism and health effects of heavy metal toxicity in humans. In: Karcioglu O, Arslan, B. (ed) Poisoning in the modern world - new tricks for an old dog, Intech Open. 10.5772/intechopen.82511

[3] Munir N, Jahangeer M, Bouyahya A, El Omari N, Ghchime R, Balahbib A, Aboulaghras S, Mahmood Z, Akram M, Ali Shah SM, Mikolaychik IN, Derkho M, Rebezov M, Venkidasamy B, Thiruvengadam M, Shariati MA (2022). Heavy metal contamination of natural foods is a serious health issue: a review. Sustainability.

[4] The drinking water directive. https://ec.europa.eu/environment/water/water-drink/legislation_en.html. Accessed 13 April 2023

[5] Safe Drinking Water Act. https://www.epa.gov/sdwa. Accessed 15 June 2023

[6] Commission Regulation (EU) 2021/1323 of 10 August 2021 amending Regulation (EC) No 1881/2006 as regards maximum levels of cadmium in certain foodstuffs. https://eur-lex.europa.eu/eli/reg/2021/1323/oj. Accessed 15 June 2023

[7] Commission Regulation (EU) 2021/1317 of 9 August 2021 amending Regulation (EC) No 1881/2006 as regards maximum levels of lead in certain foodstuffs. https://eur-lex.europa.eu/eli/reg/2021/1317/oj. Accessed 15 June 2023

[8] Bhattacharjee T, Goswami M (2018). Heavy metals (As, Cd & Pb) toxicity & detection of these metals in ground water sample: a review on different techniques. Int J Eng Sci Invention.

[9] Bulska E, Ruszczyńska A (2017). Analytical techniques for trace element determination. Phys Sci Rev.

[10] Lu Y, Liang X, Niyungeko C, Zhou J, Xu J, Tian G (2018). A review of the identification and detection of heavy metal ions in the environment by voltammetry. Talanta.

[11] Alghamdi AH (2010). Applications of stripping voltammetric techniques in food analysis. Arab J Chem.

[12] The United Nations Department of Economic and Social Affairs, The 17 goals of sustainable development. https://sdgs.un.org/goals. Accessed 15 June 2023

[13] Anastas PT (1999). Green chemistry and the role of analytical methodology development. Crit Rev Anal Chem.

[14] de Marco BA, Rechelo BS, Tótoli EG, Kogawa AC, Nunes Salgado HR (2019). Evolution of green chemistry and its multidimensional impacts: a review. Saudi Pharm J.

[15] Jovanovski V, Hočevar SB, Ogorevc B (2017). Bismuth electrodes in contemporary electroanalysis. Curr Opin Electrochem.

[16] Ariño C, Serrano N, Díaz-Cruz JM, Esteban M (2017). Voltammetric determination of metal ions beyond mercury electrodes. A review. Anal Chim Acta.

[17] Nsabimana A, Kitte SA, Fereja TH, Halawa MI, Zhang W, Xu G (2019). Recent developments in stripping analysis of trace metals. Curr Opin Electrochem.

[18] Niu X, Lan M, Zhao H, Chen C, Li Y, Zhu X (2013). Review: Electrochemical stripping analysis of trace heavy metals using screen-printed electrodes. Anal Lett.

[19] Cardoso RM, Kalinke C, Rocha RG, dos Santos PL, Rocha DP, Oliveira PR, Janegitz BC, Bonacin JA, Richter EM, Munoz RAA (2020). Additive-manufactured (3D-printed) electrochemical sensors: a critical review. Anal Chim Acta.

[20] Kokkinos C, Economou A (2020). Recent advances in voltammetric, amperometric and ion-selective (bio)sensors fabricated by microengineering manufacturing approaches. Curr Opin Electrochem.

[21] Holmes J, Pathirathna P, Hashemi P (2019). Novel frontiers in voltammetric trace metal analysis: towards real time, on-site, in situ measurements. TrAC Trends Anal Chem.

[22] Cuartero M (2012). Electrochemical sensors for in-situ measurement of ions in seawater. Sens Actuat B: Chem.

[23] Ahamed A, Ge L, Zhao K, Veksha A, Bobacka J, Lisak G (2021). Environmental footprint of voltammetric sensors based on screen-printed electrodes: an assessment towards “green” sensor manufacturing. Chemosphere.

[24] Kazmer D, Peterson AM, Masato D, Colon AR, Krantz J (2023). Strategic cost and sustainability analyses of injection molding and material extrusion additive manufacturing. Polym Eng Sci.

[25] Odularu AT (2020). Bismuth as smart material and its application in the ninth principle of sustainable chemistry. J Chem.

[26] Mohan R (2010). Green bismuth. Nat Chem.

[27] Economou A (2018). Screen-printed electrodes modified with “green” metals for electrochemical stripping analysis of toxic elements. Sensors.

[28] Messing ME (2015). The advantages of spark discharge generation for manufacturing of nanoparticles with tailored properties. J Green Eng.

[29] Meuller BO, Messing ME, Engberg DLJ, Jansson AM, Johansson LIM, Norlén SM, Tureson N, Deppert K (2012). Review of spark discharge generators for production of nanoparticle aerosols. Aerosol Sci Technol.

[30] Pfeiffer TV, Feng J, Schmidt-Ott A (2014). New developments in spark production of nanoparticles. Adv Powder Technol.

[31] Riman D, Jirovsky D, Hrbac J, Prodromidis MI (2015). Green and facile electrode modification by spark discharge: bismuth oxide-screen printed electrodes for the screening of ultra-trace Cd(II) and Pb(II). Electrochem Commun.

[32] Trachioti MG, Tzianni EI, Riman D, Jurmanova J, Prodromidis MI, Hrbac J (2019). Extended coverage of screen-printed graphite electrodes by spark discharge produced gold nanoparticles with a 3D positioning device. Assessment of sparking voltage-time characteristics to develop sensors with advanced electrocatalytic properties. Electrochim Acta.

[33] Riman D, Spyrou K, Karantzalis AE, Hrbac J, Prodromidis MI (2017). Glucose sensing on graphite screen-printed electrode modified by sparking of copper nickel alloys. Talanta.

[34] Mocak J, Bond MA, Mitchell S (1997). Scollary G (1997) A statistical overview of standard (IUPAC and ACS) and new procedures for determining the limits of detection and quantification: application to voltammetric and stripping techniques. Pure Appl Chem.

[35] Karbowska B (2016). Presence of thallium in the environment: sources of contaminations, distribution and monitoring methods. Environ Monit Assess.

[36] White SJO, Hemond HF (2012). The anthrobiogeochemical cycle of indium: a review of the natural and anthropogenic cycling of indium in the environment. Crit Rev Environ Sci Techn.

[37] Kokkinos C, Economou A, Raptis I, Efstathiou CE (2008). Lithographically fabricated disposable bismuth-film electrodes for the trace determination of Pb(II) and Cd(II) by anodic stripping voltammetry. Electrochim Acta.

[38] Gouveia-Caridade C, Pauliukaite R, Brett C (2006). Influence of nafion coatings and surfactant on the stripping voltammetry of heavy metals at bismuth-film modified carbon film electrodes. Electroanalysis.

[39] Kokkinos C, Economou A (2014). Tin film sensor with on-chip three-electrode configuration for voltammetric determination of trace Tl(I) in strong acidic media. Talanta.

[40] Directive (EU) 2020/2184 of the European Parliament and of the Council of 16 December 2020 on the quality of water intended for human consumption. https://eur-lex.europa.eu/eli/dir/2020/2184/oj. Accessed 15 June 2023

