# Supplementary material for: Eco-friendly voltammetric platform for trace metal determination using a conductive polymer sensor modified with bismuth nanoparticles generated by spark discharge
Source: Mikrochim Acta. 2023 Sep 2;190(10):376. doi: 10.1007/s00604-023-05929-2 (PMC10474985; doi:10.1007/s00604-023-05929-2)
Supplement: Supplementary file 1 — Supplementary file1 (1.06 MB) [file 604_2023_5929_MOESM1_ESM.docx]

Supplementary Material

Eco-friendly voltammetric platform for trace metal determination using a conductive polymer sensor modified with bismuth nanoparticles generated by spark discharge

Alexandra Karapa^1^, Christos Kokkinos^1^, Peter R. Fielden^2^, Sara J. Baldock^2^, Nickolas J. Goddard^3^, Anastasios Economou^1^ *, Mamas Prodromidis^4^

*^1^ Department of Chemistry, National and Kapodistrian University of Athens, Athens 157 71, Greece*

*^2^ Department of Chemistry, Lancaster University, Lancaster, LA1 4YB, UK*

*^3^ Process Instruments (UK) Ltd, March Street, Burnley, BB12 0BT, UK*

^4^ *Department of Chemistry, University of Ioannina, Ioannina*

*****Correspondence: [aeconomo@chem.uoa.gr](mailto:aeconomo@chem.uoa.gr); Tel.: +30 210 7274298


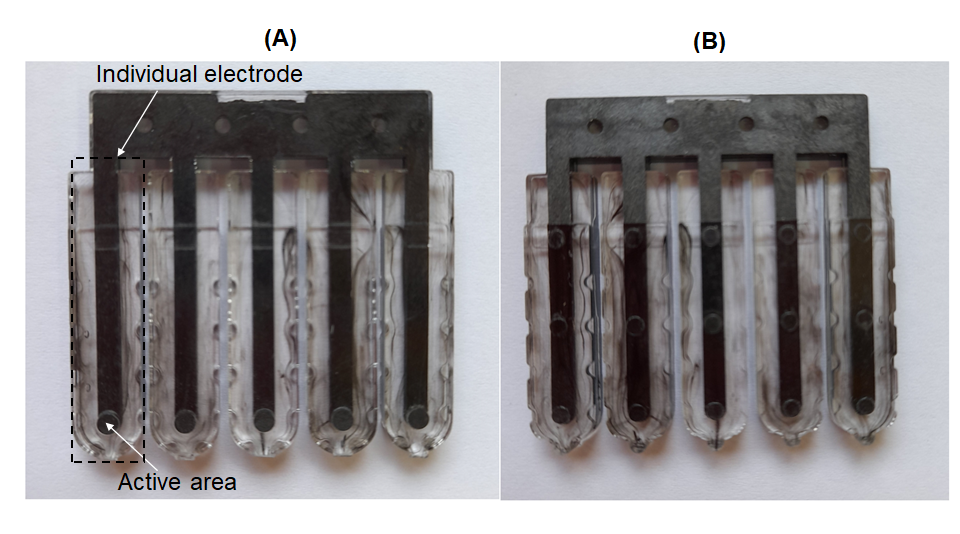


**Fig S1.** Photograph of the 5 electrode array as prepared by injection moulding. (A) top view, (B) bottom view

*Preparation of the bismuth rod for sparking*

A bismuth rod is prepared by filling a piece of borosilicate glass capillary melting point tube (1" in length, 0.05" outer diameter, Merck) open at both ends with bismuth powder (puriry 99.99 %, Sigma Aldrich) and heating at 300 ^o^ C in an oven until the bismuth melts. The bismuth-filled capillary is left to cool at room temperature until the bismuth solidifies in the form of a rod. Then, a few mm of the glass sleeve is carefully removed from both ends of the capillary to allow connection to the high-voltage power supply and contact with the substrate electrode.


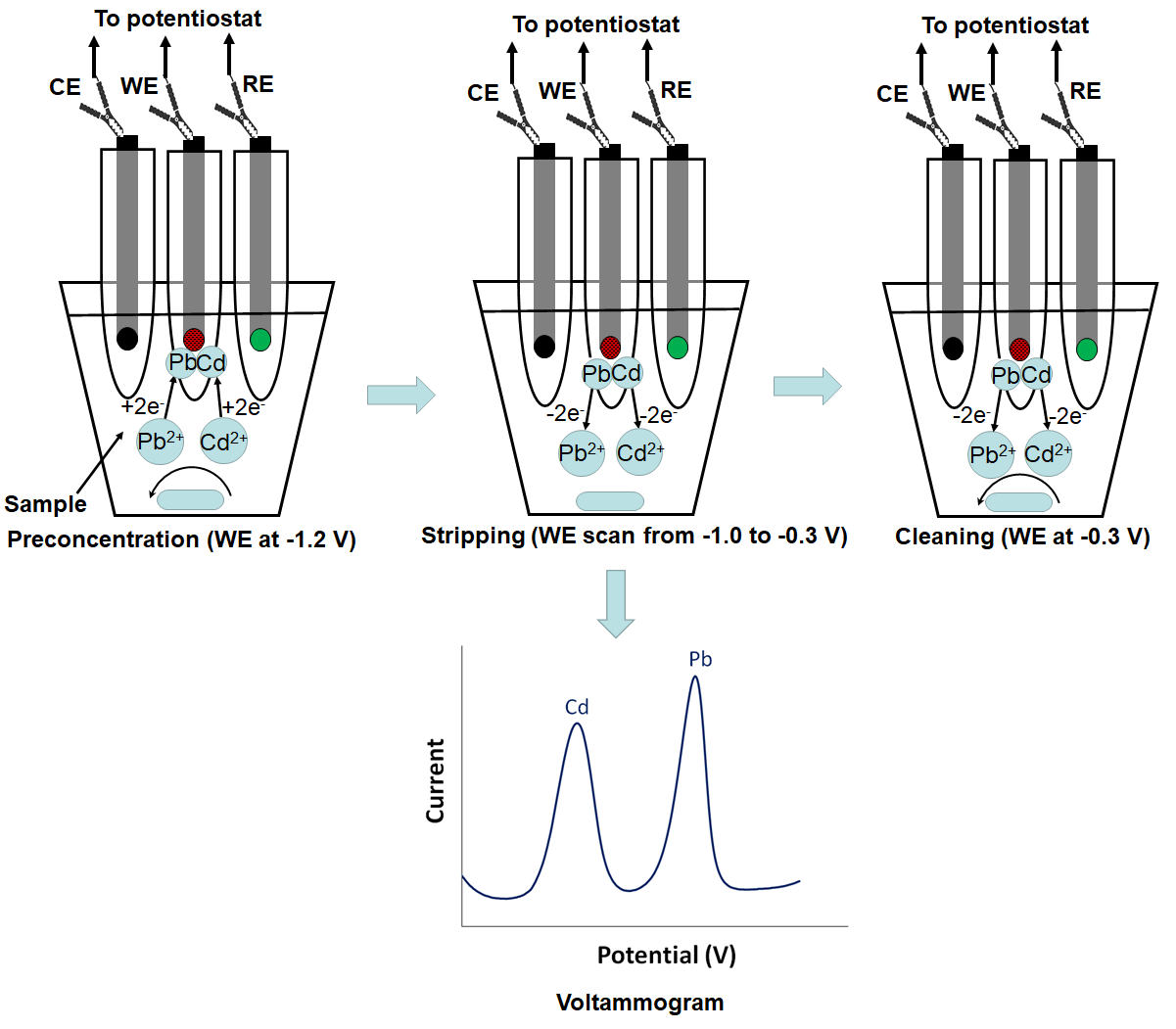


**Fig S2.** Schematic diagram of the experimental procedure for trace metal analysis using SWASV at the voltammetric platform.


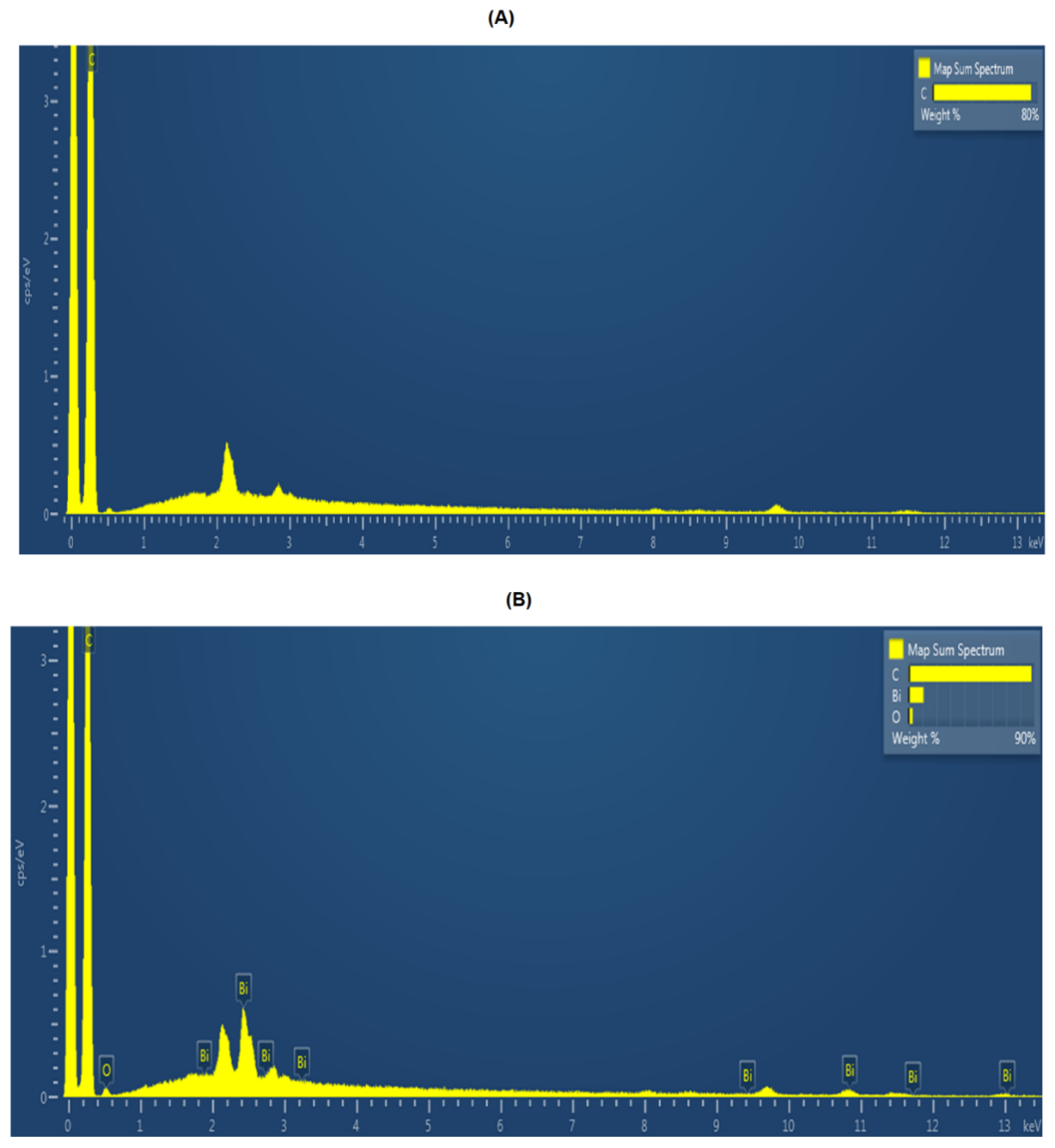


**Fig S3.** EDX spectra of (A) a bare conductive electrode and (B) a conductive electrode coated with bismuth nanoparticles using spark discharge


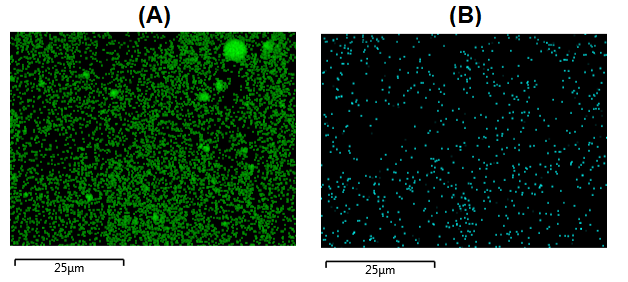


**Fig S4.** EDX mapping for (A) bismuth and (B) oxygen, at a conductive electrode coated with bismuth nanoparticles using spark discharge.


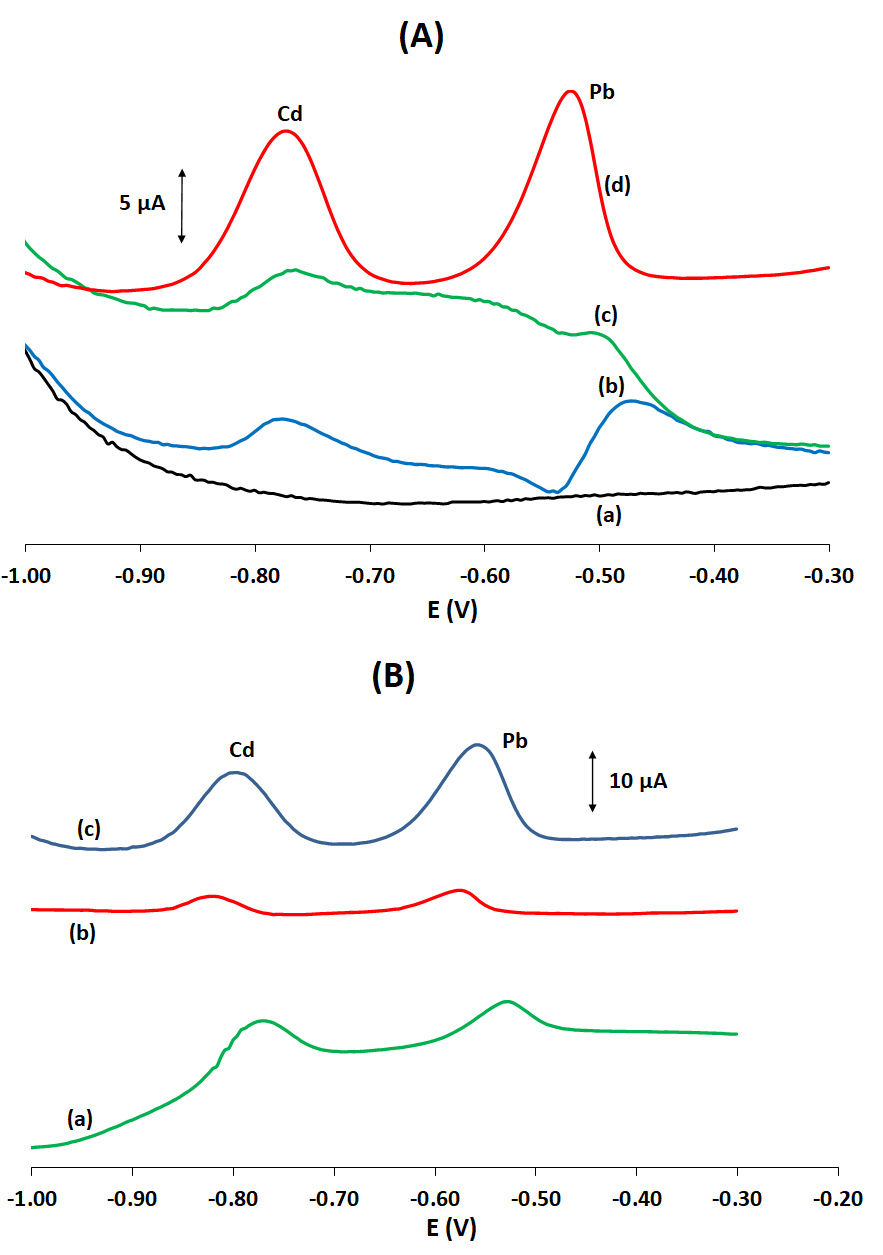


**Fig. S5**. Effect of: (A) the supporting electrolyte ((a) 0.1 mol L^-1^  H_2_SO_4_, (b) 0.1 mol L^-1^ HCl, (c) 0.1 mol L^-1^  HNO_3_ , (d) 0.1 mol L^-1^  acetate buffer (pH 4.5)) and, (B) the stripping waveform ((a) LS, (b) DP, (c) SW) on the stripping signal of 40 μg L^-1^ Pb(II) and Cd(II). LS: 100 mV s^-1^; SW: as described in the text; DP: scan rate 10 mV s^-1^; pulse amplitude, 25 mV, pulse time, 50 ms; potential step, 2 mV.


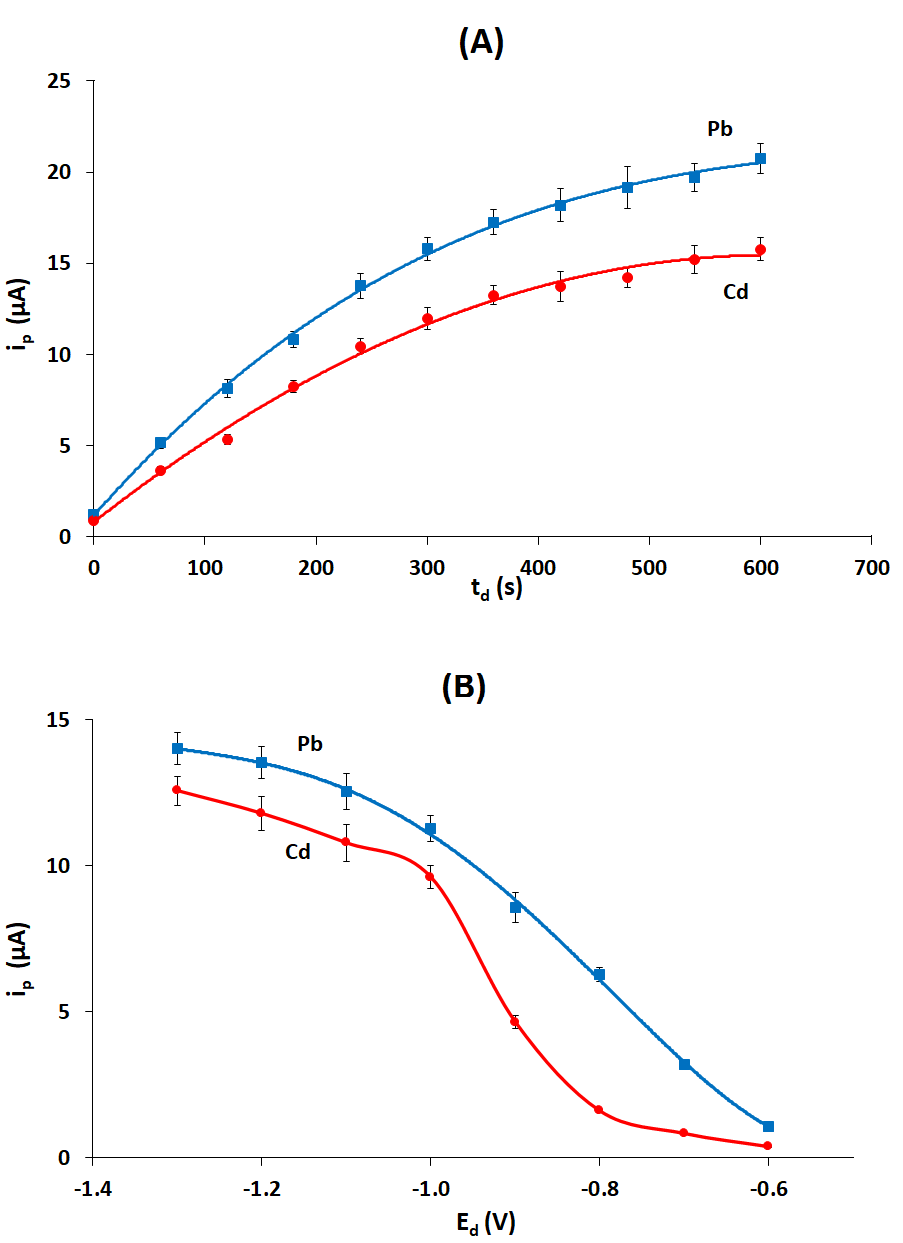


**Fig. S6.** Effect of the deposition conditions (A) deposition time, and (B) deposition potential, on the stripping peak heights of 40 μg L^-1^ Pb(II) and Cd(II) in 0.1 mol L^-1^ acetate buffer (pH 4.5). Deposition at -1.2V for 240s; other conditions as in the Experimental Part.

**Table S1**. Comparison of the bismuth-sparked injection-moulded sensors with existing sensors for the determination of Pb(II) and Cd(II).

| **Substrate** | **Limit of detection (Cd, Pb) (μg L^−1^)** | **Deposition time (s)** | **Linear range (Cd, Pb) (μg L^−1^)** | **Scope for mass fabrication** | **Modifier** | **Ref** |
| --- | --- | --- | --- | --- | --- | --- |
| **Polymer-carbon electrodes** | | | | | | |
| graphite/poly(lactic acid) | 0.3, 0.4 | 180 | 0.9-35, 1.2-35 | no | no | 1 |
| graphite/epoxy | 100, 10 | 60 | NR | no | no | 2 |
| graphite/epoxy | 1.5, 20 | NR | 5–300, 46-370 | no | Hg | 3 |
| graphite–epoxy | 7.2, 11.8 | 120 | 10–90, 10–70 | no | Bi_2_O_3_ | 4 |
| 3D-printed graphene/PLA | 2.2, 4.1 | 360 | 11-56, 16-104 | limited | Bi | 5 |
| styrene and divinylbenzene/carbon black | NR | 60-900 | NR | no | HgO | 6 |
| injection moulded polysterene/carbon fibers | 1.3, 0.95 | 240 | 4-120, 3-120 | yes | electroplated Sb | 7 |
| graphite/epoxy | 23.1, 2,8 | 120 | 15-90, 15-90 | no | Bi | 8 |
| graphite/epoxy | NR | 300 | NR | no | crown ethers | 9 |
| 3D-printed carbon black/PLA | 2.9, 2.6 | 180 | 30-270, 30-270 | limited | no | 10 |
| 3D-printed nanographite/PLA | 320, 160 | NR | 790–4500, 190–4500 | limited | no | 11 |
| 3D-printed carbon black/PLA | 11.2, 2.5 | 150 | 20-320, 20-320 | limited | no | 12 |
| graphite and poly (methyl methacrylate) | 25, 50 | 1200 | 5.6-250, 10-250 | no | no | 13 |
| **injection moulded polysterene/carbon fibers** | **0.7, 0.6** | **240** | **2-100** | **yes** | **Bi sparking** | **Present work** |
| **Disposable bismuth-modified electrodes** | | | | | | |
| pencil lead | 11, 11.5 | 250 | 48-233, 48-233 | no | electroplated Bi | 14 |
| carbon paste | 6.2, 3.4 | 180 | 224–22,400, 414-41,400 | no | Bi ferrite (bulk modification) | 15 |
| screen-printed ink | 16,8 | 120 | 20–300 | yes | Bi_2_O_3_ (bulk modification) | 16 |
| screen-printed ink | 1.1, 0.9 | 120 | 10–80, 5–50 | yes | Bi_2_O_3_ and bismuth salts (bulk modification) | 17 |
| screen-printed ink on paper | 1,1 | 240 | 5–150 | yes | electroplated Bi | 18 |
| screen-printedgraphene/polyaniline/polystyrene nanoporous fibers | 4.4, 3.3 | 180 | 0–500 | yes | electroplated Bi | 19 |
| silicon | 1, 0.5 | 120 | 10–90, 5–45 | yes | sputtered Bi | 20 |
| graphene paper | 0.1,0.1 |  | 5–100, 5-100 | limited | Nafion-Bi by ion exchange | 21 |
| screen printed ink | 0.5, 0.8 | 150 | 1-60, 1-60 | yes | reduced graphene oxide modified in situ with Bi | 22 |
| inkjet-printed ink | 1.1-67, 2-124 |  | 0.6, 1.5 | yes | Bi (drop casting) | 23 |
| gold ultramicroelectrode array | 7, 5 | 600 | 20-100, 10-80 | yes | electroplated Bi | 24 |


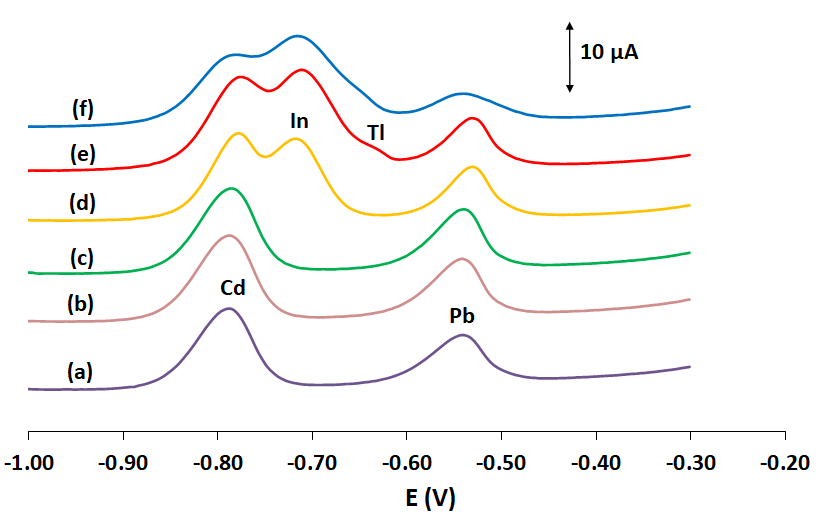


**Fig. S7.** Interference study. SW stripping voltammograms of: (a) 40 μg L^-1^ Cd(II) and 20 μg L^-1^ Pb(II), (b) as (a) with 40 μg L^-1^ Sn(II), (c) as (b) with 40 μg L^-1^ Sb(III), (d) as (c) with 40 μg L^-1^ In(III), (e) as (d) with 40 μg L^-1^ Tl(I), (f) as (e) with 40 μg L^-1^ Cu(II). Supporting electrolyte, 0.1 mol L^-1^ acetate buffer (pH 4.5); deposition at -1.2 V for 240 s; other conditions as in the Experimental Part.

**References**

# [Silva](https://pubs.rsc.org/en/results?searchtext=Author%3AAna%20Lu%C3%ADsa%20Silva) AL, [Corrêa](https://pubs.rsc.org/en/results?searchtext=Author%3AMatheus%20Millen%20Corr%C3%AAa) MM, [de Oliveira](https://pubs.rsc.org/en/results?searchtext=Author%3AGrasielli%20Correa%20de%20Oliveira) GC, [Michel](https://pubs.rsc.org/en/results?searchtext=Author%3ARicardo%20Cunha%20Michel) RC, [Semaan](https://pubs.rsc.org/en/results?searchtext=Author%3AFelipe%20Silva%20Semaan) FS, [Ponzio](https://pubs.rsc.org/en/results?searchtext=Author%3AEduardo%20Ariel%20Ponzio) EA (2018) Development and application of a routine robust graphite/poly (lactic acid) composite electrode for the fast simultaneous determination of Pb^2+^ and Cd^2+^ in jewelry by square wave anodic stripping voltammetry. New J Chem 42:19537-19547.

1. Carrégalo S, Merkoçi A, Alegret S (2004) Application of Graphite-Epoxy Composite Electrodes in Differential Pulse Anodic Stripping Voltammetry of Heavy Metals. Microchim Acta 147**:**245–251 (2004).
2. Fernández LL, Bastos-Arrieta J, Palet C, Baeza, M (2022) Composite Electrodes Based on Carbon Materials Decorated with Hg Nanoparticles for the Simultaneous Detection of Cd(II), Pb(II) and Cu(II). Chemosensors 10:148.
3. Castaneda MT, Perez M, Pumera M, del Valle M, Merkoci A, Alegret S (2005) Sensitive stripping voltammetry of heavy metals by using a compositesensor based on a built-in bismuth precursor. Analyst 130: 971–976
4. Walters JG, Ahmed S, Terrero Rodríguez IM, O'Neil GD (2020) [Trace Analysis of Heavy Metals (Cd, Pb, Hg) Using Native and Modified 3D Printed Graphene/Poly(Lactic Acid) Composite Electrodes](https://analyticalsciencejournals.onlinelibrary.wiley.com/doi/10.1002/elan.201900658). Electroanalysis *32:*859-866.

# [Seo](https://pubs.acs.org/action/doSearch?field1=Contrib&text1=Kyoungwon++Seo) K, Kim S, Park, J (1998) Modified Composite Electrode Containing HgO as Built-In Mercury Precursor for Anodic Stripping Analysis of Trace Metals. Ana*l. Chem.* 70: 2936–2940

1. Christidi S, Chrysostomou A, Economou A, Kokkinos C, Fielden P.R, Baldock SJ, Goddard NJ (2019) Disposable Injection Molded Conductive Electrodes Modified with Antimony Film for the Electrochemical Determination of Trace Pb(II) and Cd(II). Sensors 19: 4809.
2. Kırgoçz AU, Marín S, Pumera M, Merkoci A, Alegret S (2005) Stripping Voltammetry with Bismuth Modified Graphite-Epoxy Composite Electrodes. Electroanalysis 17:881-886.
3. González-Calabuiga A, Guerrero D, Serrano N, del Valle M (2016) Simultaneous voltammetric determination of heavy metals by use
   of crown ether-modified electrodes and chemometrics. Electroanalysis 28: 663-670.
4. Rocha DP, Squissato AL, da Silva SM, Richter EM, Munoz RAA (2020) Improved electrochemical detection of metals in biological samples using 3D-printed electrode: Chemical/electrochemical treatment exposes carbon-black conductive sites. Electrochim Acta 335:135688.
5. Foster CW, Elbardisy HM, Down MP, Keefe EM, Smith GC, Banks CE (2020) Additively manufactured graphitic electrochemical sensing platforms. Chem Eng J 381: 122343.
6. de Oliveira FM, de Melo EI, da Silva RAB (2020) 3D Pen: A low-cost and portable tool for manufacture of 3D-printed sensors. Sens Actuat B: Chemical 321:128528,
7. Perween M, Srivastava DN (2017) [A Cost-Effective, Unmodified Platform for the Detection of Heavy Metals via Anodic Stripping Voltammetry at Nanomolar Level](https://chemistry-europe.onlinelibrary.wiley.com/doi/10.1002/slct.201700477) ChemistrySelect 2: 4428.
8. Bedin KC, Mitsuyasu EY, Ronix A, Cazetta AL, Pezoti O, **Almeida VC (2018)** Inexpensive Bismuth-Film Electrode Supported on Pencil-Lead Graphite for Determination of Pb(II) and Cd(II) Ions by Anodic Stripping Voltammetry. **Intern J Anal Chem** 2018:1473706.
9. [Beyene](https://pubs.rsc.org/en/results?searchtext=Author%3AYonas%20Beyene) Y, [Bitew](https://pubs.rsc.org/en/results?searchtext=Author%3AZelalem%20Bitew) Z,  [Fekade](https://pubs.rsc.org/en/results?searchtext=Author%3AFasika%20Fekade) F. (2022) Electrochemical detection of Pb(II) and Cd(II) using bismuth ferrite nanoparticle modified carbon paste electrodes. **Mater Adv 3:**5882-5892.
10. Kadara RO, Tothill IE (2008) Development of disposable bulk-modified screen-printed electrode based on bismuth oxide for stripping chronopotentiometric analysis of lead (II) and cadmium (II) in soil and water samples. Anal Chim Acta 623:76-81.
11. Lezi Ν, Economou A, Dimovasilis PA, Trikalitis PN, Prodromidis MI (2012) Disposable screen-printed sensors modified with bismuth precursor compounds for the rapid voltammetric screening of trace Pb(II) and Cd(II). Anal Chim Acta 728:1–8.
12. Rattanarat P, Dungchai W, Cate D, Volckens J, Chailapakul O, Henry CS (2014) Multilayer Paper-Based Device for Colorimetric and Electrochemical Quantification of Metals. Anal Chem 86:3555–3562.
13. Promphet N, Rattanarat P, Rangkupan R, Chailapakul O, Rodthongkum N (2015). An electrochemical sensor based on graphene/polyaniline/polystyrene nanoporous fibers modified electrode for simultaneous determination of lead and cadmium. Sens. Actuat. B Chem. 207:526–534.
14. Kokkinos C, Economou A, Raptis I, Efstathiou CE (2008) Lithographically fabricated disposable bismuth-film electrodes for the trace determination of Pb(II) and Cd(II) by anodic stripping voltammetry. Electrochim Acta 53: 5294-5299
15. Scandurra A, Ruffino F, Urso M, Grimaldi MG, Mirabella S (2020). Disposable and Low-Cost Electrode Based on Graphene Paper-Nafion-Bi Nanostructures for Ultra-Trace Determination of Pb(II) and Cd(II). Nanomaterials. 10:1620.
16. Ping J, Wang Y, Wu J, Ying Y (2014) Development of an electrochemically reduced graphene oxide modified disposable bismuth film electrode and its application for stripping analysis of heavy metals in milk. Food Chem 151:65–71.
17. Guenang L, Gupta P, Basseto V, Jovic M, Ymélé E, Lesch A, Girault H, Tonlé I (2020) Oxygen Plasma/Bismuth Modified Inkjet Printed Graphene Electrode for the Sensitive Simultaneous Detection of Lead and Cadmium. Americ J Anal Chem 11**:**1-14.
18. Bahinting SED, Rollon AP, Garcia-Segura S, Garcia VCC, Ensano BMB, Abarca RRM, Yee JJ, de Luna MDG (2021) Bismuth Film-Coated Gold Ultramicroelectrode Array for Simultaneous Quantification of Pb(II) and Cd(II) by Square Wave Anodic Stripping Voltammetry. Sensors 21:1811.
